# Willingness of Tea Farmers to Adopt Ecological Agriculture Techniques Based on the UTAUT Extended Model

**DOI:** 10.3390/ijerph192215351

**Published:** 2022-11-21

**Authors:** Kexiao Xie, Yuerui Zhu, Yongqiang Ma, Youcheng Chen, Shuiji Chen, Zhidan Chen

**Affiliations:** 1 Anxi College of Tea Science, Fujian Agriculture and Forestry University, Quanzhou 362406, China; 2 College of Horticulture, Fujian Agriculture and Forestry University, Fuzhou 350002, China; 3 Fujian Anxi Tieguanyin Tea Science and Technology Backyard, Quanzhou 362406, China; 4 Anxi Agricultural and Rural Bureau, Quanzhou 362400, China; 5 Engineering Technology and Research Center of Fujian Tea Industry, Fuzhou 350002, China

**Keywords:** ecological agricultural technology, ecological tea plantation, unified theory of technology adoption and use, perceived value, Wuyishan city, Anxi county

## Abstract

Ecological agricultural technology is the key method for making the transition from traditional agriculture to ecological agriculture, and is also the basic measure for promoting the transformation and upgrading of the tea industry and sustainable development. This study explores the influencing factors and mechanisms of tea farmers’ adoption of ecological agricultural technology by using the extended model of the unified theory of technology adoption and use (UTAUT) based on perceived value. The analysis results, using the partial least squares structural equation model (PLS-SEM), show that: the positive impact of perceived value on willingness to use not only makes the explanatory power of the extended model greater than that of the original model but also expands the UTAUT model into a full mediating model, in which performance expectation has the greatest impact on behavioral intention through the implemented value. Effect expectation, social influence and factoring factors following, then the four intermediary paths have significant positive effects on behavioral intention. This study improves on the limitations of the UTAUT theoretical model through the theory of perceived value, and provides a reference for research on the same topic. At the same time, the government should provide tea farmers with enhanced subsidies, skills training and communication platforms.

## 1. Introduction

China is the largest tea producer in the world and has a long history of planting and cultivating tea. The tea industry has a high economic value, and it is the economic source of livelihood for many farmers [1]. Most tea plantations rely on traditional management methods that use a high level of chemical input, including fertilizers, pesticides, and herbicides, to maintain their output of tea. This has led to a series of problems such as soil erosion, soil acidification, pesticide residues, and reduced biodiversity [2,3], posing a serious threat to the environment, crops, and personal safety. To alleviate the disadvantages of traditional agricultural management and coordinate the relationship between human needs and protection of the natural environment, the concept of ecological agriculture has been proposed from the field of ecological economics [4]. It emphasizes the role of intercropping, landscape transformation, green prevention and control, and the application of organic fertilizers, among other measures, in reducing external input in agriculture and maximizing the regulatory function of the natural environment. Thus, ecological agriculture can help to protect the ecological environment in production and to move ecological agriculture technology closer to the concept and related technical definitions of sustainable agriculture, green agriculture, and organic agriculture [5].

Studies have shown that the adoption of sustainable ecological agricultural technologies contributes to improvements in environmental carrying capacity and system stability [2], while also improving the quality of the tea produced [3]. However, the practical effects of ecological agricultural technology are often not immediate, especially given the outdated knowledge reserve of many tea farmers and their weak awareness of ecological environment protection [6,7]. Once they understand the risk costs of technology transfer and the use of new technologies, tea farmers often have little interest in adopting eco-agricultural technologies with long-term benefits [8]. To encourage the adoption of such technologies, it is therefore necessary to explore the driving factors for tea farmers. Danne and Musshoff [9] argued that farmers hoped to obtain additional premium income and reduce feed input through ecological grazing, and that good infrastructure of pasture plays a role in promoting the adoption of eco-agricultural technology. Zhu and Chen [10] found that the greater the availability of technical support, policy subsidies, and environmental publicity, the more farmers tend to adopt green agricultural production technology. According to the field survey of Verbeke et al. [11], most livestock farmers perceived more benefits than risks in terms of the effects of insect feed; the main appeal was that insect feed can increase the nutritional value of feeding animals, which is a sustainable, low-cost, and high-profit feeding method.

In summary, while the existing literature does not lack investigation into farmers’ intentions to adopt eco-agriculture technology from various angles, the existing research is not based on a mature and systematic theoretical framework; thus, the overall theoretical framework and the explanatory power of the research are weak, and the persuasiveness of the influencing factors revealed by the research needs to be further improved [12]. Although the unified theory of technology adoption and use (UTAUT) provides a systematic framework, and some studies have already applied it to the field of farmer technology adoption [13,14,15,16,17,18,19], most research has focused on major food crops or animal husbandry, paying little attention to tea farmers. Different from grains and meat, tea is not a necessary agricultural cash crop for people’s daily life. If the use of ecological agricultural technology leads to an increase in production costs, it may reduce the overall income of tea farmers. For this reason, tea farmers are most concerned about the expected costs and benefits after the adoption of ecological agricultural technology, rather than food safety, environmental damage, and other issues. Therefore, this study expands the original model by introducing perceived value as the intermediary variable, so as to improve the theoretical framework of the study. On the one hand, this study enriches the literature on tea farmers in the field of technology acceptance, and puts forward some targeted policy recommendations. On the other hand, by introducing the variable of perceived value, this study has formed a new theoretical model, which provides a theoretical reference for researchers of other similar issues.

## 2. Theoretical Basis and Research Hypothesis

### 2.1. Unified Theory of Technology Adoption and Use (UTAUT)

**Figure 1:**
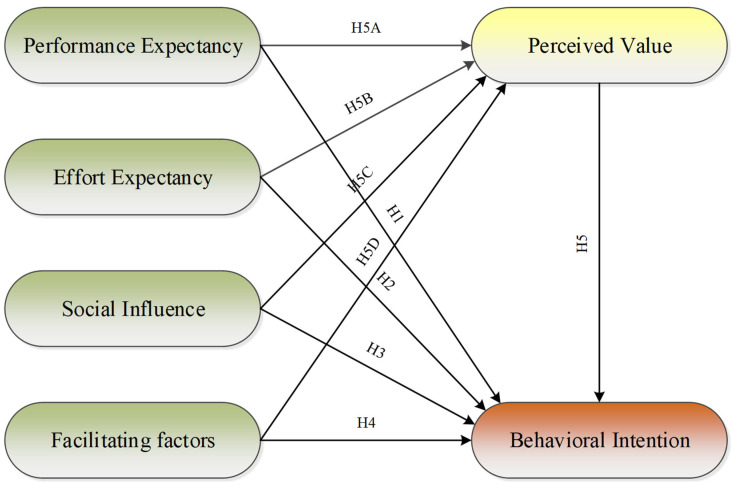
Conceptual framework.

UTAUT was proposed by Venkatesh and Davis [12] in 2003 to analyze research on the adoption of information technology. Reviewing eight classical adoption theories (the task-technology fit model, the diffusion of innovation theory, the theory of rational action, the theory of planned action, the technology adoption model, the theory of planned action model, the motivational model, and social cognitive theory), they integrated the arguments and extracted four core constructs that affect willingness to adopt new technology: (1) performance expectation, the degree to which individuals expect to be helped by the use of the technology; (2) effort expectation, the level of effort required by individuals to use the technology; (3) social influence, the degree to which an individual’s social environment affects their use of the technology; and (4) facilitating factors, the degree to which the internal and external conditions of the individual’s expected use of the technology promote their use of the technology. UTAUT is widely accepted in the field of social research because of its explanatory power (up to 70%) [12]. In recent years, in the field of agriculture, UTAUT has been widely applied to the Internet of Things [19,20], communication technology [21,22], and mobile applications [23,24], among other areas of technology-adoption intention research. However, UTAUT theory has rarely been applied in the study of tea farmers’ willingness to adopt ecological agricultural technologies. In order to better understand the psychological mechanisms in play, this study introduces perceived value into the UTAUT model as a mediating variable. The extended model not only provides support for work in related fields but is also suitable for exploring the influence mechanism of perceived value on the adoption of ecological farming technology. Figure 1 shows the conceptual framework of this study.

### 2.2. Research Hypotheses

In the context of this study, performance expectation is the extent to which tea farmers believe they can benefit from using ecological agricultural techniques in tea plantations [12]. Performance expectation, which includes expectations of ecological benefits, economic benefits, and social benefits [17,25,26], is one of the factors that directly and effectively predict whether tea farmers will adopt ecological agricultural technology. When they believe that the use of ecological agricultural technology will produce higher benefits, their willingness to adopt that technology is greater. Effort expectation is the cost and effort that tea farmers think is required to master ecological agricultural technology in tea gardens [12]. When they believe that learning a new technology is relatively easy and does not add much cost in money or time, they are more inclined to try it [27,28].

Social influence is the extent to which public opinion and the views and practices of important people affect the adoption of ecological agricultural technology in tea plantations by tea farmers [12]. From the perspective of social networks and subjective norms, a large number of studies have demonstrated that the views and practices of family and friends affect the technical identity of tea farmers due to the so-called neighborhood effect [29]. Therefore, community publicity and online media opinion can influence adoption intentions by presenting a social consensus and generating public opinion pressure [30]; the degree and the effects of peer use also have an impact [31,32]. The more positive the views conveyed by society, the higher the willingness of tea farmers to adopt the technology.

Facilitating factors are the influence of the degree of support provided to tea farmers for the implementation of ecological agricultural technology [12]. In tea gardens, the application of fertilization and management technology requires farmers to reach a certain threshold of relevant knowledge and investment of money. Professional training and policy subsidies can mitigate the effect of this threshold and improve the willingness of tea farmers to adopt ecological agricultural technology [33,34].

The theory of perceived value was initially applied to the intentions and behavior of consumers [35]. Woodruff [36] proposed a hierarchical model of three cognitive differences in perceived value, distinguishing three dimensions: basic attitude; meaning cognition; and value recognition. Ren et al. and Li and Chen [37,38] later applied this classification to farmers’ behavior, demonstrating its significant explanatory power in relation to the behavior and final adoption intentions of farmers. In sum, the greater the perceived value of a behavior, the more likely tea farmers are to adopt that behavior. Technology adoption behavior is a type of economic decision; accordingly, tea farmers take on the role of rational economic agents pursuing the maximization of benefits [39], and a change in any factor can affect their perception of technical value, which is composed of a variety of benefits and costs. Thus, tea farmers’ willingness to adopt a particular technology depends on their perception of the value of benefits leading to a comprehensive decision.

The following hypotheses are proposed in this study.

**Hypothesis** **1** **(H1).** *Performance expectation has a positive impact on the intention to adopt ecological agricultural technologies in tea gardens;*

**Hypothesis** **2** **(H2).** *Effort expectation has a positive impact on the intention to adopt ecological agricultural technologies in tea gardens;*

**Hypothesis** **3** **(H3).** *Social influence has a positive impact on the intention to adopt ecological agricultural technologies in tea gardens;*

**Hypothesis** **4** **(H4).** *Promoters have a positive impact on the intention to adopt ecological agricultural technologies in tea gardens;*

**Hypothesis** **5** **(H5).** *Perceived value has a positive impact on the intention to adopt ecological agricultural technologies in tea gardens;*

**Hypothesis** **5A** **(H5A).** *Perceived value plays a mediating role between performance expectation and the intention to adopt ecological agricultural technologies in tea gardens;*

**Hypothesis** **5B** **(H5B).** *Perceived value plays a mediating role between effort expectation and the intention to adopt ecological agricultural technologies in tea gardens;*

**Hypothesis** **5C** **(H5C).** *Perceived value plays a mediating role between social influence and the intention to adopt ecological agricultural technologies in tea gardens;*

**Hypothesis** **5D** **(H5D).** *Perceived value plays a mediating role between facilitating factors and the intention to adopt ecological agricultural technologies in tea gardens.*

## 3. Materials and Methods

### 3.1. Questionnaire Design

This study used a questionnaire consisting of two parts, one for demographic characteristics and one that formed the core of the data collection. The core included four explanatory variables, namely performance expectation, effort expectation, social influence, and facilitating factors; the mediating variable, namely perceived value; and the explained variable, namely tea farmers’ willingness to adopt technology, which was itself composed of six factors [1,12,15,16,37,38]. The final form of the questionnaire was based on the existing literature and took into account expert guidance on the characteristics of the survey objects from the Department of Tea Science and the Department of Business and Economics of Fujian Agriculture and Forestry University. Appropriate adjustments were made in light of pre-survey feedback. Each variable in the core part of the questionnaire was represented by four to six items, and the items were rated on a five-point Likert scale (1 = strongly disagree, 2 = disagree, 3 = neither agree nor disagree, 4 = agree, 5 = strongly agree). The core part of the formal questionnaire is reproduced in Appendix A.

### 3.2. Data Collection

From June to September 2022, questionnaire responses were collected in Fujian Province, which has the largest tea output in China. The questionnaires were distributed in Wuyishan City of Fujian Province and Anxi County of Quanzhou City of Fujian Province, the birthplace of Wuyi rock tea and Tie Guanyin tea. Face-to-face interviews, telephone surveys, and internet surveys were conducted. The interviewees in this article were randomly selected. We set three criteria for interviewees: First, the interviewees must be aborigines in the area we were investigating. Secondly, respondents’ family members must have tea gardens. Third, the respondents were engaged in tea-related work. To balance cost and effectiveness and to obtain the most accurate and reliable data possible, this study includes questionnaire responses both from the face-to-face surveys and from targeted distribution of online questionnaires (each method accounting for approximately 50% of responses). Respondents were asked to read the notes carefully before responding to the questionnaire to ensure that they would understand the questionnaire content and to avoid issues with definitions of ecological agricultural technology.

### 3.3. Data Analysis

The Smart-PLS 3.0 application was used to model the structural equation and analyze the survey results. Exploratory factor analysis was used to determine the reliability and validity of the research model and the fitting index. Confirmatory factor analysis was used to determine the relationships between variables and test the research hypotheses. The mediating effect test was then applied to determine the size and type of the mediating effect of perceived value in the extended model.

## 4. Results

### 4.1. Sample Characteristics

A total of 178 valid completed questionnaires were received, with males accounting for 70.2% of responses and participants aged 31–50 years accounting for 52.8%. In terms of education, 40.4% had technical secondary school or high school education, and 44.9% had college-level education or higher. This indicates that the main managers of these tea gardens have a certain level of knowledge, which may reflect the fact that the young and middle-aged labor force who benefited from China’s compulsory education have gradually inherited the management of the tea gardens from their older predecessors. Table 1 gives the demographic characteristics of the sample.

Table 2 shows the descriptive statistics. The values for performance expectation (M = 4.560, SD = 0.564), effort expectation (M = 3.904, SD = 0.849), social influence (M = 4.324, SD = 0.620), promoting factors (M = 4.337, SD = 0.634), and perceived value (M = 4.349, SD = 0.605) have high mean values and low standard deviations indicating that tea farmers are optimistic about ecological agricultural technology as a whole (M = 4.394, SD = 0.620), and are sensitive to all factors that promote or hinder the use of ecological agricultural technology.

### 4.2. Reliability and Validity

Table 3 shows the reliability and validity analyses. For all measurement dimensions, Cronbach’s alpha coefficient (CA) and the composite reliability (CR) values were both greater than 0.8, the average variance extracted (AVE) was greater than 0.6, and the variance inflation factor (VIF) was greater than 0.2 but less than 5. This indicates that the measurement model has good internal consistency [40] and convergent validity [41] and that there are no multicollinearity problems [42]. The Fornell and Larcker AVE test was applied to verify the discrimination effectiveness of the measurement items by cross-loading [43]. As Table 4 shows, the square root value of the AVE of all measurement dimensions is greater than the square correlation value of the reflection factor and the dimension in the same row on the left, which indicates good structural discrimination.

### 4.3. Hypothetical Path Testing

**Figure 2:**
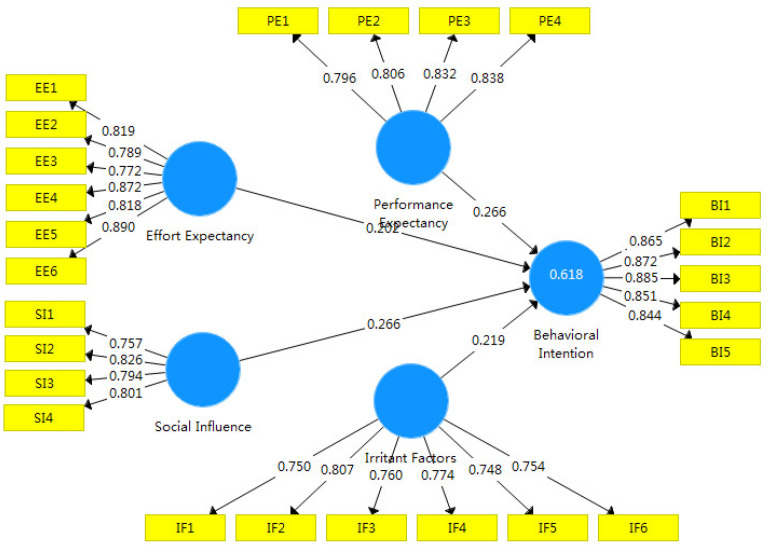
Original UTAUT model.

**Figure 3:**
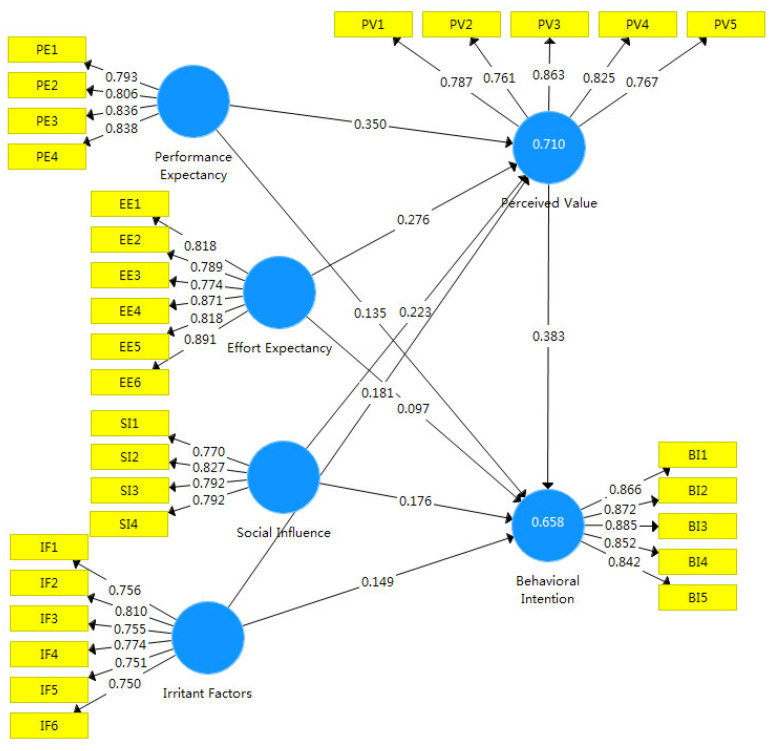
Extended UTAUT model.

In order to test the impact path differences before and after the introduction of perceived value into the UTAUT model, this study analyzed and compared the indicators of the original UTAUT model (Model 1) and the extended version (Model 2) (Figure 2 and Figure 3, respectively). Table 5 shows that the SRMR values for Models 1 and 2 were all less than 0.080, the d_ULS values were all less than 3, and the NFI values were all greater than 0.6, which indicates that both models passed the fit test and that the analysis results are valid. The R^2^ of Model 2 (0.658) is 0.050 greater than the R^2^ of Model 1 (0.618), which confirms that Model 2 has greater explanatory power and that the introduction of perceived value was appropriate.

In Model 1, performance expectation, effort expectation, social influence, and promoting factors all had significant positive effects on the willingness of tea farmers to adopt ecological agricultural technology (*p* < 0.01). Performance expectation (effect = 0.266) and promoting factors (effect = 0.266) had the greatest impact, and therefore hypotheses H1, H2, H3, and H4 are supported in Model 1. In Model 2, performance expectation, effort expectation, social influence, and promotion factors were not significantly related to tea farmers’ intentions to adopt ecological agricultural technology (*p* > 0.10), and therefore hypotheses H1, H2, H3, and H4 are not supported in Model 2. However, the four explanatory variables mediated by perceived value had a significant positive influence on the path of tea farmers’ intentions to adopt ecological agricultural technology (*p* < 0.05), and therefore hypotheses H5, H5A, H5B, H5C, and H5D are supported. This shows that farmers are very concerned about the increase in costs after the adoption of technology, resulting in a decrease in benefits. When farmers feel that the adoption of ecological agricultural technology can improve efficiency without paying too much effort and money costs, they are very willing to use this technology.

Table 6 shows the four mediation paths obtained using the BootStrap (*n* = 5000) autonomous sampling method (performance expectation 95%CI = (0.230, 0.447), effort expectation 95% CI = (0.143, 0.444), social influence 95%CI = (0.062, 0.423), and promoting factors 95%CI = (0.055, 0.297). The influence of four paths on adoption intention through perceived value did not contain 0 between the upper and lower limits of the 95% confidence interval, which indicates that the mediating effects of the four paths are all significant. The mediating effect of performance expectation (effect = 0.350) was the most significant; in other words, the extended model became a full mediating model after the introduction of perceived value. According to this research result, it is verified that when tea farmers adopt ecological agricultural technology, they mainly focus on the changes in benefits and costs after the adoption of technology, rather than a series of potential problems, such as social relations, social reputation, or environmental protection.

## 5. Discussion

This study, drawing on UTAUT and the definition of perceived value, introduced perceived value as an intervening variable. The results in the original model for performance expectation, effort expectation, social influence, and promoting factors of tea farmers’ ecological agriculture technology adoption intentions are significant and positive, as they are in the extended model, Through the mediating effect of perceived value, performance expectation, effort expectation, social influence, and promotion factors all had significant positive correlations with tea farmers’ intentions to adopt ecological agricultural technology; however, the direct effect on adoption intentions was not significant. This indicates that whether tea farmers are willing to adopt ecological agricultural technology depends on the usefulness of the technology, its perceived ease of use, the influence of people around them, and the stimulus of external factors [1,18,44]. However, the mediating role of perceived value should not be ignored [35,36]. The possible reasons for perceived value’s role as a complete mediating variable are clear. When tea farmers perceive that they can obtain improvements in effectiveness and convenience from ecological agricultural technology, their value judgments and positive attitude toward the technology are improved and their intention to adopt is enhanced.

On the basis of this analysis, four policy recommendations can be made. First, when governments promote ecological agricultural technology, they should strengthen policy subsidies and promotion, as well as formulate policy measures to help tea farmers reduce the burden of technology costs and improve their incomes [45,46]. It important to understand and apply the appropriate policies to a higher degree, because even if governments issue appropriate policies, tea farmers may not be aware of the developments.

Second, technical training and informative assistance are required. Compared with other occupations, tea farmers generally have a low level of education, and most technical descriptions of ecological agricultural technical measures are difficult to understand. Misunderstandings may therefore lead to improper applications of the technology, which would be counterproductive. The involvement of scientific and technical personnel at grassroots level, as well as regular training courses [13,47] to help tea farmers correctly grasp the applications of new technology, would be conducive to continuous use and word-of-mouth promotion of technology.

Third, by supporting a group of farmers to demonstrate how to run ecological tea gardens, governments could take advantage of the neighborhood effect among farmers. Demonstration tea gardens supported by preferential policies and subsidies [48] would help to establish a reliable social network platform for tea farmers, providing a smooth information channel for them to exchange experiences, and popularizing the application of the technology from area by area [49].

Fourth, governments should focus on propagating the value of technology by means of frequent media coverage of relevant technical knowledge and environmental protection information. Daily exposure to such information would improve tea farmers’ awareness of ecological environment protection and lead to a more accurate understanding of ecological agricultural technology, thereby engendering a positive attitude toward ecological agricultural technology [50,51].

## 6. Conclusions

Drawing on a UTAUT model extended by the introduction of perceived value, this study explored the factors that influence tea farmers’ intention to adopt ecological agricultural technology. The analysis shows that performance expectation, effort expectation, social influence, facilitating factors, and perceived value had significant positive effects on the intention to adopt. Perceived value played a full mediating role, and the extended model used in this study had a stronger explanatory power than the original model. In other words, the assumptions H1, H2, H3, and H4 in this study are demonstrated in the original model 1 of UTAUT. After the introduction of perceived value, the assumptions H5, H5A, H5B, H5C, and H5D are supported in the extended model 2 of UTAUT. This not only confirmed the correctness of the relationship logic analyzed in the research hypothesis, but also concluded that the change in expected income and cost is the problem of the most concern for tea farmers. The government can reduce the various costs of tea farmers using ecological agricultural technology and improve the use of ecological agricultural technology by tea farmers. The results of the study indicate that it is necessary to strengthen policy support, technical support, and social network construction to improve the dissemination of ecological agricultural technology. This study makes three main contributions. First, the research findings on the factors that influence the willingness of tea farmers to adopt ecological agriculture technology enrich the theoretical support in the field of tea garden management, and provide suggestions and references for the in-depth promotion of tea-garden ecological agriculture technology. Second, the introduction of perceived value as a mediating variable in the UTAUT framework provides a theoretical model with a higher explanatory power that clarifies the psychological mechanism affecting the adoption of ecological agricultural technology by tea farmers. Third, the results of this study provide a basis for policy suggestions that can help the government to formulate and promote appropriate ecological transformation policies for local tea plantations. In addition, this study has three main limitations that should be noted. First, although the introduction of perceived value improves the explanatory power of the model and enriches the application of UTAUT in the field of tea farmer technology adoption, further improvements are possible. The theory of perceived value is based on the individual’s trade-off between benefits and costs, which is divided into two aspects: perceived gains and perceived losses [35]. However, this study, in the same way as previous research in the area, focused on perceived gain and neglected perceived loss. Although it is because the existing research does not provide reliable references, it is undeniable that future research should include perceived loss to obtain a more systematic and powerful explanatory model. Second, the demographic characteristics of the participants in this study are not fully representative of tea farmers. Fujian Province is an economically developed coastal province, and the education level of tea farmers there is higher than in the country as a whole (and higher than in developing countries generally). Future research should seek to improve the generalizability of the findings by expanding the sample collection to include rural grassroots areas. Third, many tea farmers have multiple occupations, and it is likely that this is an important factor in their willingness to adopt new technology. However, the research methods of the present study are not suited to analysis of that specific point, and we would urge future research to remedy this.

## Figures and Tables

**Table 1:** Sample characteristics.

Variable	Definition	Frequency (*n*)	Proportion (%)
Gender	Male	125	70.2
	Female	53	29.8
Age	20–30 years	62	34.8
	31–40 years	54	30.3
	41–50 years	40	22.5
	51–60 years	15	8.4
	Older than 60 years	7	4.0
Educational level	Elementary school and below	3	1.7
	Junior high school	23	12.9
	Technical secondary or high school	72	40.4
	College or bachelor’s degree	62	34.8
	Graduate and above	18	10.1
Occupation	Renting land to others and working in a tea company	36	20.2
	Engaged in agriculture during the production season, working outside the home the rest of the time	60	33.7
	Mostly farming, with occasional outside work to supplement household income	31	17.4
	Full-time farming	51	28.7

**Table 2:** Descriptive statistics.

Variable	Number of ITEMS	Mean	Standard Deviation
Performance expectation	4	4.560	0.564
Effort expectation	6	3.904	0.849
Social influence	4	4.324	0.620
Facilitating factors	6	4.337	0.634
Perceived value	5	4.349	0.605
Behavioral intention	5	4.394	0.620

**Table 3:** Scale reliability.

Variable	Item	Factor Loading	AVE	CR	CA	VIF	Reliability/ Validity Criteria
Performance expectation	PE1	0.793	0.670	0.890	0.836	1.621	using
	PE2	0.806				1.819	
	PE3	0.836				2.060	
	PE4	0.838				1.958	
Effort expectation	EE1	0.818	0.685	0.929	0.908	2.180	using
	EE2	0.789				2.182	
	EE3	0.774				2.074	
	EE4	0.871				2.876	
	EE5	0.818				2.305	
	EE6	0.891				3.360	
Social influence	SI1	0.770	0.632	0.873	0.806	1.695	using
	SI2	0.827				1.943	
	SI3	0.792				1.688	
	SI4	0.792				1.606	
Facilitating factors	IF1	0.756	0.587	0.895	0.860	1.923	using
	IF2	0.810				2.198	
	IF3	0.755				1.799	
	IF4	0.774				2.400	
	IF5	0.751				2.391	
	IF6	0.750				1.626	
Perceived value	PV1	0.787	0.643	0.900	0.860	1.901	using
	PV2	0.761				1.768	
	PV3	0.863				2.588	
	PV4	0.825				2.158	
	PV5	0.767				1.759	
Behavioral intention	BI1	0.866	0.745	0.936	0.915	2.686	using
	BI2	0.872				2.897	
	BI3	0.885				3.132	
	BI4	0.852				2.517	
	BI5	0.842				2.427	

**Table 4:** Fornell and Larcker test.

Variable	BI	EE	IF	PV	PE	SI
Behavioral intention	0.863					
Effort expectancy	0.584	0.828				
Irritant factors	0.637	0.467	0.766			
Perceived value	0.769	0.655	0.642	0.802		
Performance expectancy	0.632	0.465	0.466	0.705	0.818	
Social influence	0.718	0.589	0.757	0.746	0.639	0.795

Note: BI: Behavioral Intention; EE: Effort Expectancy; IF: Irritant factors; PV: Perceived value; PE: Performance Expectancy; SI: Social Influence, the same below.

**Table 5:** Model path test comparison.

Path Hypothesis		Model 1			Model 2		
		Effect of Value	*p*	Decision	Effect of Value	*p*	Decision
PE–BI	Direct	0.266	0.000 **	Accept	0.135	0.085	Reject
EE–BI	Direct	0.202	0.00 **	Accept	0.097	0.134	Reject
SI–BI	Direct	0.266	0.007 **	Accept	0.176	0.060	Reject
FF–BI	Direct	0.219	0.005 **	Accept	0.149	0.063	Reject
PV–BI	Direct				0.383	0.000 **	Accept
PE–PV–BI	Indirect				0.350	0.000 **	Accept
EE–PV–BI	Indirect				0.276	0.000 **	Accept
SI–PV–BI	Indirect				0.223	0.010 *	Accept
FF–PV–BI	Indirect				0.181	0.004 **	Accept
R^2^		0.618			0.658		
SRMR		0.069			0.070		
d_ULS		1.558			2.302		
Chi-square		733.343			1057.123		
NFI		0.770			0.742		

Note: * represents that the path assumption has a significant impact relationship at the level of 5%, ** represents that the path assumption has a very significant impact relationship at the level of 1%.

**Table 6:** Mediating effect test.

Path Hypothesis	Effect of Value	95% Confidence Interval		Result
		LLCI	ALSO	
PE–PV–BI	0.350	0.230	0.447	Mediation
EE–PV–BI	0.276	0.143	0.444	Mediation
SI–PV–BI	0.223	0.062	0.423	Mediation
FF–PV–BI	0.181	0.055	0.297	Mediation

## Data Availability

The data presented in this study are available within the article.

## Appendix A. Survey Instrument

All questions were rated on a five-point Likert scale (1 = strongly disagree, 2 = disagree, 3 = neither agree nor disagree, 4 = agree, 5 = strongly agree).

Performance expectations:

PE-1: The adoption of ecological agricultural techniques will lead to an increase in crop yield or quality.

PE-2: The products produced by the use of ecological agricultural technology are more popular in the market and the price is higher.

PE-3: The adoption of ecological agricultural technology can improve soil fertility.

PE-4: The adoption of ecological agricultural technology can increase the environmental vegetation and improve the ecological environment.

Effort expectation

EE-1: I can master ecological farming techniques easily through learning.

EE-2: The total cost of using ecological technology is lower than that of traditional production methods.

EE-3: At least the adoption of ecological agricultural technology will not reduce my yield.

EE-4: I have enough time and energy to use eco-farming techniques.

EE-5: I can overcome the technical threshold or barrier of applying ecological agricultural technology.

EE-6: I do not find it difficult to transition from traditional agricultural technology to ecological agricultural technology.

Social influence:

SI-1: I will adopt ecological farming techniques that most of the farmers around me use.

SI-2: When large farmers or enterprises demonstrate and achieve good technical results, I will be willing to adopt them.

SI-3: If the government gives a call and promises to subsidize it, I am willing to adopt it.

SI-4: I will respond to the request of cooperatives to adopt agroecological technologies.

Facilitating factors:

FF-1: The lower the price of outsourcing services for eco-agricultural technology, the greater my willingness to adopt agroecological technology.

FF-2: I will be more willing to use agricultural machinery and equipment with low pollution if it can provide preferential treatment.

FF-3: Fertilizer subsidies with organic fertilizer, no pollution, and low toxicity will increase my willingness to use.

FF-4: Whether the technical training of professionals is provided free of charge will affect whether I adopt agroecological technology.

FF-5: The number of visits and attitudes of agricultural technology training personnel will affect my willingness to adopt agroecological technology.

FF-6: The better the guidance effect of agricultural technology trainers, the more willing I am to use them.

Perceived value:

PV-1: In general, I am positive about the adoption of eco-agricultural technologies.

PV-2: Generally speaking, I have a good understanding of the significance of ecological agricultural technology in protecting the environment and preventing and controlling pollution.

PV-3: Generally speaking, I think ecological agricultural technology has more environmental and economic value.

PV-4: I believe that the total benefits of adopting ecological technologies can cover the total costs of adopting them.

PV-5: Compared with labor, I think it is cost-effective to invest energy in learning the use of ecological agricultural technologies.

Willingness to adopt ecological agricultural technologies (behavioral intention)

BI-1: I am willing to adopt eco-agricultural technologies into my own production practices.

BI-2: I am willing to learn the technical knowledge of ecological agriculture under the guidance of agricultural technicians.

BI-3: If demonstrations by others or my own planting work well, I am willing to recommend it to relatives, friends, or people around me.

BI-4: I am willing to try new ecological farming techniques when they are promoted.

BI-5: I will continue to use the ecological agricultural technologies that have been adopted in the future.
